# Root-inspired, template-confined additive printing for fabricating high-robust conformal electronics

**DOI:** 10.1038/s41378-024-00840-z

**Published:** 2024-12-14

**Authors:** Guifang Liu, Xiangming Li, Yangfan Qiu, Chuanhang Zeng, Xinkai Zhu, Chao Wang, Xiaoliang Chen, Chunhui Wang, Hongmiao Tian, Jinyou Shao

**Affiliations:** 1 Micro- and Nano-technology Research Center, State Key Laboratory for Manufacturing Systems Engineering, Xi’an Jiaotong University, Xi’an, Shaanxi 710049 China; 2 Frontier Institute of Science and Technology (FIST), 28 Xianning Road, Xi’an Jiaotong University, Xi’an, Shaanxi 710049 China

**Keywords:** Electrical and electronic engineering, Electronic devices

## Abstract

Conformal electronic devices on freeform surface play a critical role in the emerging smart robotics, smart skins, and integrated sensing systems. However, their functional structures such as circuits tend to tear-off, break, or crack under mechanical or thermal influence when in service, thus limiting the application reliability of conformal electronics. Herein, inspired by the tree root system, template-confined additive (TCA) printing technology was presented for reliable fabrication of robust circuits. TCA printing technology involves the penetration of adhesive into the functional material, thereby enhancing the mechanical robustness of the circuits, allowing them to maintain their electrical performance despite the presence of external damaging factors such as scratching, abrasion, folding, and high temperatures. For example, herein, the circuits could withstand mechanical abrasion at temperatures as high as 350 °C without compromising electrical properties. Benefiting from the confines of template, the printed circuits achieved resolutions of up to 300 nm, suitable for various materials such as P(VDF-TrFE), MWCNTs, and AgNPs, which enabled the multi-material self-aligned fabrication. Furthermore, the versatility of TCA printing was presented by fabricating circuits on arbitrary substrates, and realizing various devices, such as conformal temperature/humidity sensing system and epidermal ultra-thin energy storage system. These applications present the significant potential of TCA printing in fabricating intelligent devices.

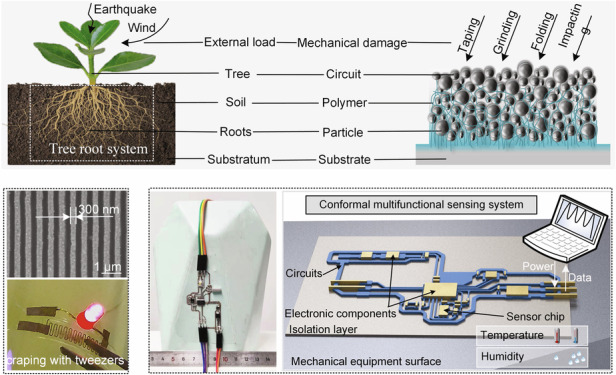

## Introduction

The effective coupling of functional circuits with three-dimensional (3D) device geometries has generated many emerging application scenarios, such as smart skin of the aircraft^1–3^, smart bearing^4,5^, hemispherical electronic eye lenses^6,7^, non-planar antenna^8,9^, and smart manipulators^10,11^. Although a variety of printing techniques has been used to fabricate 3D electronics, the printed electronics are usually attached to the substrate due to surface energy only and they exhibit poor mechanical stability, thus they can get easily damaged under normal usage in service environments. For instance, functional structures such as circuits tend to tear-off, break, or crack when subjected to mechanical or thermal influences, thus affecting the desired functionality of the circuit^12^ and limiting the potential applications of conformal electronics. Furthermore, high-performance multifunctional conformal electronics also demand high resolution, precision, and multi-material adaptability^13,14^, rendering the efficient fabrication of such circuits challenging due to the inherent limitations of existing manufacturing technologies.

Typical ink-printing technology is the primary technique for constructing 3D graphics, which involves the direct manufacturing of circuits on substrates with the assistance of computer-aided engineering and shows high flexibility. The representative technologies of 3D printing include extrusion printing^15,16^, inkjet printing^17^, aerosol jet printing^18^, and electrohydrodynamic printing^19^. These printing approaches, although promising, suffer from some inherent limitations. The first and foremost of these limitations is the adhesion issue of printed circuits. The adhesion of the functional material to the substrate is influenced by the combination of the adhesive energy of the ink particles and the surface energy of the substrate. The simple stacking of these heterogeneous materials (ink and substrate) creates weakly bonded interfaces (even if their adhesion is improved by hydrophilic treatment of the substrate), making it challenging for circuits to exhibit strong mechanical robustness. This leads to potential issues, in particular, in operational environments beyond the laboratory stage, where circuits may experience external mechanical damage (such as scratches, impacts, etc.) or temperature fluctuations (high and low temperatures), resulting in peel-off, detachment, or cracks, thereby causing electronic malfunctions^20^. Furthermore, the functional materials for printing are usually slurry-based inks, and when the ink is printed onto the substrate, it flows laterally due to its inherent rheological wettability^21^, which inevitably destroys the originally designed circuit resolution and reduces accuracy. Furthermore, the performance of 3D-printed conformal structures is heavily dependent on the equipment configuration, where the resolution, accuracy, and path precision of printed circuits are directly proportional to the cost of the equipment^22^. It indicates that the achievement of high-quality conformal electronics entails increased manufacturing costs.

To date, different strategies have been developed to address these issues, for instance, in terms of enhancing robustness, more mechanically durable circuits have been prepared by infiltrating conductive polymers into fiber frameworks^23,24^, or by partially embedding conductive particles within the substrate^25,26^. However, circuits printed by such methods often fall short in terms of versality, and it remains a challenging task to simultaneously attain both high precision and high resolution, thus making the realization of high-performance circuits difficult. In terms of improving circuit resolution and accuracy, the transfer of intricately pre-fabricated 2D patterns onto 3D substrates has proven to be a promising approach for realizing high-quality circuitry prints^27^. The typical techniques include 3D elastic rubber transfer printing^28^, hydro printing^29^, and selected adhesion transfer printing^30,31^. Moreover, methods such as stretching^32^ or folding^33,34^ have been employed to wrap the flexible graphic around the target substrate to form a 3D electronic. Although these transfer techniques inherit the advantages of fabrication of 2D electronics to achieve high-performance 3D electronics, the bonding form of conductive materials stacked on the substrate introduces intrinsic adhesion defects, which makes it difficult to maintain the optimal electron performance under the influence of temperature, scratches, impacts, etc.^35,36^. In the emerging novel applications, higher demands are placed on the fabrication of conformal electronics^37^. Mechanical robustness of electronics such as circuits, is a fundamental prerequisite for environmental adaptability. However, high-quality manufacturing, characterized by high resolution and precision, is central to improving electronic performance; nonetheless, these requirements increase manufacturing challenges.

**Fig. 1:**
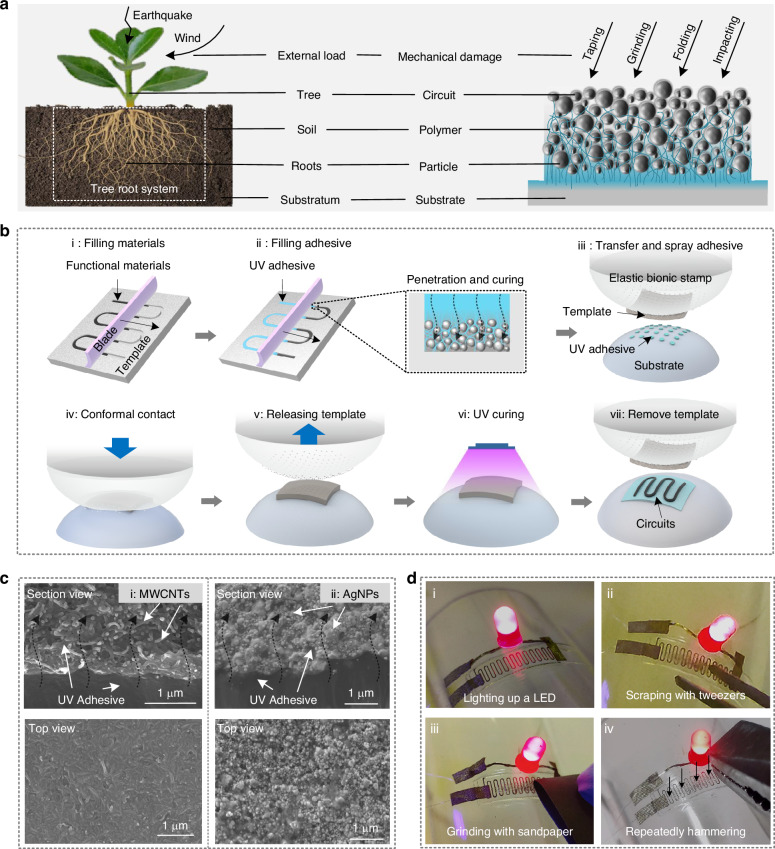
TCA printing. **a** Schematic illustration of the analogy between a tree against the natural load and the circuits against the mechanical loads. **b** Fabrication process of the TCA printing. **c** SEM images of the section and top view of the printed MWCNTs and Ag circuits. **d** Demonstration of strong robustness of printed circuits. When the LED is lighted using the printed serpentine circuit, it still lights up when the circuit is subjected to tweezers scraping, sandpaper grinding, and repeated hammered, respectively

In terms of mechanistic studies on mechanically robust structures, one well-established example that nature has been demonstrating since time immemorial is the mechanism of a tree root system to support the load of trees^38^. This naturally formed reliable foundation allows the root system to withstand various loads, such as wind, earthquake, and its self-weight, without failure. The extensive root system penetrates the soil, promoting tight stacking of the soil particles and a connection with the substratum, thus reinforcing their ability to resist loads and ensuring the stability of the tree^39^. Drawing inspiration from the load-bearing mechanism of tree roots, this study developed a mechanically robust circuit capable of infiltrating polymers into conductive particles (Fig. 1a). The circuit and the polymer are analogous to the tree and the soil, the lower circuit particles are analogous to tree roots, the substrate is analogous to a substratum layer, and the external mechanical load is equivalent to the natural load of violent winds or earthquakes. The polymer binds the circuit to the substrate in depth, forming a robust structure that can withstand various mechanical loads.

Specifically, herein, a simple, effective, and reliable fabrication technique called Template-Confined Additive (TCA) printing was proposed for printing conformal circuits with strong robustness and high resolution. The TCA printing technique involves shape-defining of the circuit using a flexible confined template, thus strengthening the circuit with adhesive penetration. It utilizes a bionic elastic stamp to print the pre-fabricated circuit onto a 3D substrate. The TCA printing technology is primarily used for solution-processible materials and shows similarities to other stamp printing in terms of operating procedures and technical characteristics (detailed comparative analysis is provided in Supporting information Table S1). However, its uniqueness lies in the ability to strengthen the mechanical robustness of printed circuits, enabling them to demonstrate reliability in various tests, such as taping, high temperatures, scratching, and folding, thus possessing broad potential prospects in harsh environments. The TCA technique enables in-situ fabrication of high-quality conformal electronics with a resolution of up to 300 nm, thus it is suitable for a wide range of materials (silver nanoparticles (AgNPs), multi-walled carbon nanotubes (MWCNTs), polyvinylidene difluoride (PVDF), etc.), and capable of achieving self-aligned printing of multiple layers of materials. Moreover, the TCA printing technology tolerates a wide range of receiving substrates with various surface morphologies (smooth/rough substrates) and materials (ceramics, glass, magnesium alloys, acrylics, etc.). Demonstrations of the integrated temperature/humidity sensing system and conformal ultra-thin energy storage system produced by TCA printing on equipment surfaces indicate the potential of this technology in developing lightweight and intelligent equipment for innovative device applications.

## Results and discussion

### TCA printing strategy

Figure 1b shows schematic illustration of TCA printing process of an AgNPs-based circuit. The process involves the preparation of a functional graphic in a flexible template (filled with functional material and adhesive, and then cured), picking up the flexible template using an elastic bionic stamp, and pressing prefabricated graphic conformal contacts onto a 3D substrate. The functional graphic is transferred to the substrate by secondary curing polymer, and the empty flexible template is then removed. The flexible template was fabricated by photolithography and vacuum molding processes (Fig. S1), inheriting the nanoscale precision and resolution of the silicon process. The functional graphic can be fabricated by various filling strategies, such as scrape coating^40^, printing^41^, and microfluidics^42^, as long as the materials remain filled into the template and do not interconnect at the surface. The elastic bionic stamp, used as a printing media, was prepared by fabricating a bionic adhesion layer on the surface of an elastic stamp (Fig. S2). The elastic bionic stamp (Fig. S3) was selected herein because of the following two major reasons: (1) the ability to reliably pick up and release the template, and (2) the ability of flexible to fit a variety of shapes of curved receptor substrates due to their elastic deformability, and the corresponding details are presented in Fig. S4. Figure S5 shows the customized TCA printing equipment, exhibiting that the elastic bionic stamp can move back and forth to reach a specified workstation, and can also move up and down to enable pickup/release of a template or conformal pressing on a receiving substrate. Moreover, in the proposed method, first the ink was dried to form a conductive structure and then the voids were filled with polymers, unlike mixing conductive particles with polymers and then curing them (Fig. S6). This difference leads to better conductivity of the proposed method. Figure 1c illustrates the cross-sectional and top views of the printed MWCNTs and Ag circuits. Evidently, the ultraviolet (UV) adhesive gets infiltrated into the interior of the conductive particles, establishing a strong bond with the conductive particles at the interface, and even penetrating the top. This profound connection endows the circuit with robust mechanical integrity. Figure 1d shows an example of strong mechanical robustness of a printed circuit; i.e., a serpentine circuit printed on an acrylic cylindrical surface that lights up a light-emitting diode (LED). When the circuit is subjected to various external damages, such as being scraped with tweezers, ground with sandpaper, or repeatedly hammered, the circuit remains conductive and the LED continues to emit light (Movie S1).

### Robust characterization of printed circuits

**Fig. 2:**
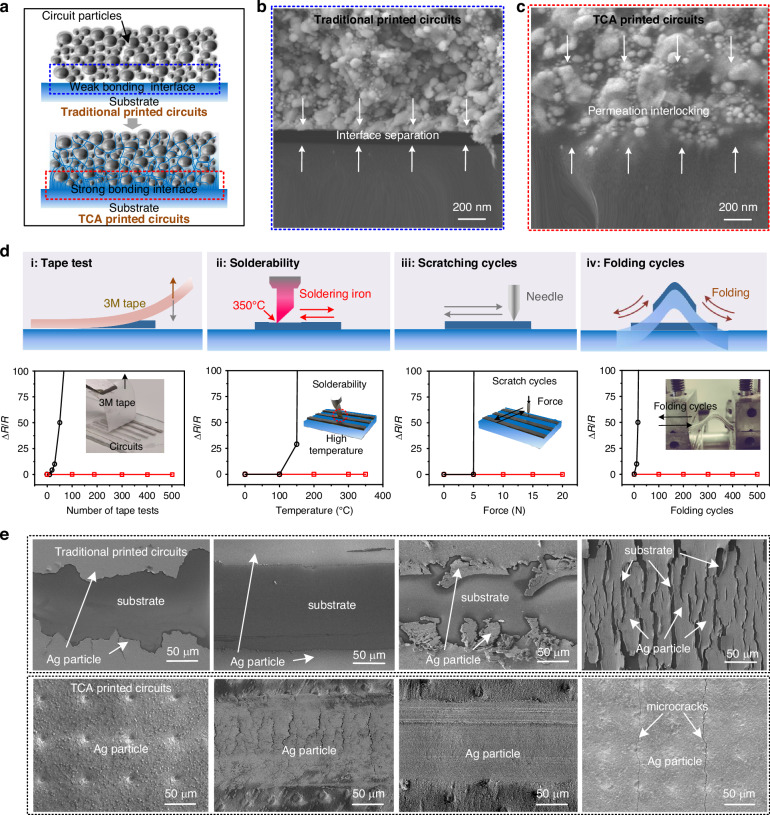
Characterization of the adhesion stability of printed circuits. **a** Schematic of cross-section of traditionally printed circuit (top) and TCA-printed circuit (bottom). **b** Cross-section SEM image of a traditionally printed circuit. **c** Cross-section SEM image of a TCA printed circuit. **d** Testing of traditionally printed circuits and TCA-printed circuits for taping, solderability, scratching, and folding, respectively, schematic (top) and resistance comparison of circuits after testing (bottom), and (**e**) SEM images presenting comparison of the morphology of the circuits after testing

Conformal circuits, such as sensing cells affixed to the exterior of aircraft or robots, require exceptional robustness to maintain operational stability in extreme environments^43,44^. The proposed TCA printing technology triumphs over traditional printing methods in terms of robust attachment, and the schematic of the underlying principle is shown in Fig. 2a. In traditionally printed circuits, the functional material particles are directly attached to the substrate, and the particle–substrate contact interface is weakly bonded. In TCA-printed circuits, the functional material particles are embedded in the adhesive, which results in a deeply penetrated interlocking interface that ensures a robust bond. To demonstrate this, circuits fabricated on glass substrates via both traditional and TCA printing techniques were immersed in liquid nitrogen for 2 min and subsequently fractured to expose their cross-sections. Figure 2b evidently illustrates that the Ag-particle material in traditionally printed circuits exhibits poor bonding with the substrate, resulting in cracks and detachment of the Ag circuits following exposure to the extremely cold liquid nitrogen. Conversely, Fig. 2c illustrates that in TCA-printed circuits, the UV adhesive penetrates deeply into the Ag particles, ensuring a robust bond. Even when exposed to the freezing temperatures of liquid nitrogen, the interface between the UV adhesive and the Ag particles remains stably bonded. Moreover, excellent adhesive properties of the UV adhesive lead to its firm bonding onto the glass substrate.

The electrical properties of the material are significantly influenced by the morphology of the functional material due to the penetration of the adhesive into the material gap during the printing process. Four representative functional materials, including eutectic gallium–indium (EGaln), AgNPs, silver nanowires (AgNWs), and poly(3,4-ethylenedioxythiophene) (PEDOT), were printed into lines with a width of 200 μm and a length of 2 cm, respectively. The resistance values were tested before and after printing, and the results are shown in Figure S7. The circuit before TCA printing is the one within the PDMS template (the circuit printed in the channel), and its electrical properties are similar to those of traditionally printed circuits (the circuit printed on a flat surface). Therefore, the difference in resistance performance before and after TCA printing is the disparity between traditionally printed circuits and circuits printed by TCA. The results show that the resistance characteristics of EGaIn, AgNPs, and PEDOT hardly change after printing, while the resistance of AgNWs and MWCNTs increases after printing. The common characteristic shared by EGaln, AgNPs, and PEDOT is their dense structure, which leads to the shallow penetration depth of the polymer during printing. This shallow penetration preserves the contact between electrically conductive particles, maintaining stable electrical properties. Considering Ag inks as an example, it was found that nano-Ag inks formed effective conductive networks when cured in the templates, which minimized the contact resistance between particles, resulting in an excellent resistivity of 5.8 × 10^−7^ Ω·m for printed circuits. This value is superior to that of ordinary printed circuits^45,46^, but slightly lower than the resistivity of bulk Ag due to inter-particle interfacial resistance and potentially incomplete densification regions. Conversely, the circuit morphology of the AgNWs and MWCNTs exhibits a stack of wire materials that form larger pores, which facilitates deeper penetration of the polymer. This deeper infiltration might induce slight displacement of the nanowire materials during the curing process of the UV adhesive, potentially resulting in incomplete contact between conductive nanowires and consequently, an increase in resistance.

For quantitative evaluation of the robustness of printed circuits, the TCA-printed and conventionally-printed circuits were subjected to a series of rigorous tests, including taping tests, high-temperature tests (solderability), scratch cycles, and folding cycles, respectively, and the results are shown in Fig. 2d and e. First, the stability of the circuits was tested by the tape method (first column of Fig. 2d,e). In this method, 3 M tape was firmly pressed onto the circuits, ensuring complete contact with the conductive material, and then the tape was torn off vertically to test the peel resistance of the circuits. Quantitative test results show that traditionally printed circuits failed completely after about 50 tape tests, while TCA-printed circuits did not produce any changes and maintained stable resistance even after being subjected to 500 tape tests. The SEM images prove that the Ag particles were completely stripped from the substrate in the conventionally-printed circuits; however, no change occurred in the TCA-printed circuits.

The ability of active devices to be soldered to circuits is important for the development of conformal electronics, thus the high-temperature tests (solderability) of the circuit were also performed (second column of Fig. 2d,e). A soldering iron was set to a specific temperature and scraped across the circuits, and then the resistance performance was recorded. At temperatures above 200 °C, the resistance of conventionally-printed circuits increased rapidly, due to the detachment of electrical particles from the substrate during high-temperature scratching. By contrast, TCA-printed circuits exhibited stable resistance even at temperatures of 350 °C. The SEM image shows the circuits to be more tightly stacked and partially compressed into the melted polymer at high-temperature scratches, with slight cracking along the direction of the scratches. This resistance stability at high-temperature scratching meets the basic requirements for soldering active devices.

Equipment attached with surface conformal electronics may get scratched by external objects in operation, thus the stable operation of the circuit should be ensured under such conditions. Therefore, scraping tests were also performed. In the scraping cycle test (third column of Fig. 2d,e), the circuits were repeatedly scraped with a needle tip by applying pressure, and the electrical properties and morphology of the traditionally printed and TCA-printed circuits were comparatively analyzed. The scratch cycle tests reveal that when the pressure exceeded 5 N, the resistance of the traditionally printed circuits surged, leading to circuit failure. However, the TCA-printed circuits demonstrated remarkable stability, maintaining consistent resistance even under a scratching pressure of 20 N. The SEM images show that when a pressure of 10 N was applied, the Ag particles of the traditionally printed circuits were easily scraped off the substrate by the needle tip, resulting in complete circuit separation and subsequent failure. However, the Ag particles of the TCA-printed circuits were found to be compressed and flattened while remaining securely adhered to the substrate, ensuring stable resistance and continuous functionality.

Moreover, the folding test was conducted to demonstrate the robustness performance of the printed electronics in flexible applications (fourth column of Fig. 2d,e). The circuits with a width of 2 mm and length of 20 mm were fabricated on polyethylene terephthalate (PET) substrates by conventional printing and TCA printing techniques, respectively. The circuits were fixed on a ball-screw platform for bending tests to measure resistance changes, followed by observation of their morphology after 500 cycles. The results show that the resistance of traditionally-printed circuits increased sharply at around 50 cycles, while the TCA-printed circuits demonstrated remarkable stability, with their resistance remaining unchanged even after 500 cycles. Visually, the traditionally printed circuits showed prominent cracks at the folding area, with Ag particles curling or detaching, fragmenting the Ag circuits into disconnected islands. By contrast, the TCA-printed circuits exhibited only minor surface cracks without any loss of Ag particles, ensuring superior electrical conductivity. Incidentally, due to the limited tensile properties of the UV-cured adhesive used herein, the tensile properties of the circuits printed by TCA also suffered restriction. Figure S8 presents the test results of the tensile properties of TCA-printed circuits. The results show that cracks gradually appear in the circuit when it is subjected to tensile force until it breaks. Nevertheless, owing to the infiltration of UV adhesive into the circuit, compared with circuits printed by traditional methods, TCA printing augments the tensile resistance of the circuit.

Given that UV adhesives act as external load distributors, the selection of UV adhesives is crucial for determining the mechanical properties of printed circuits. Table S2 and Figure S9 present a comparative illustration of typical different types of UV adhesives when the circuit is subjected to mechanical loads. The results prove that the preferred NOA71 exhibits more comprehensive robust performance in TCA printing. Moreover, owing to the different adhesion of NOA71 adhesive to different material substrates, experiments on taping and scratching were performed on circuits printed on a variety of substrates (Fig. S10). The results show that NOA possesses excellent adhesion on most substrates (glass, copper foil, PET film, filter paper, and acrylic plate), which is mainly attributed to the roughness or high surface energy characteristics of these substrates. For smooth and low-surface-energy materials such as Teflon films or sheets, most adhesives, and not just NOA71, cannot strongly adhere to the substrate unless this type of substrate is treated with oxygen plasma to enrich the surface with hydrophilic groups, which can enhance the adhesion between the adhesive and the substrate.

### Morphological characterization of printed circuits

**Fig. 3:**
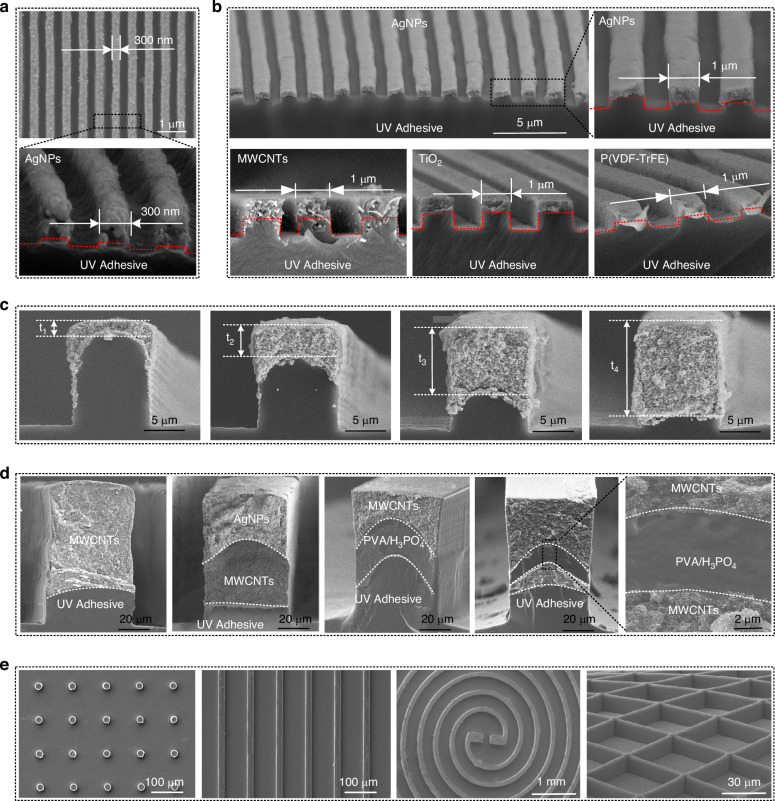
Demonstration of printing results. **a** SEM image of top view (top) and cross-section (bottom) of printed AgNPs grids with a line width of 300 nm. **b** SEM images of printed AgNPs, MWCNTs, TiO_2_, and P(VDF-TrFE) materials with a line width of 1 μm. **c** SEM images of different thicknesses of printed circuits. **d** SEM images of printed circuits with different layers. **e** SEM images of printed circuits with different morphologies

The templates used in TCA printing play a crucial role in defining the circuit shapes and morphology, enabling functional materials to exhibit superior precision and resolution within its confines. The theoretical resolution achievable with TCA printing technology is primarily determined by the minimum line width of the template, yet it is also affected by the size of the material particles. For instance, the particles are more easily accommodated within the confines of template only when the particle size is significantly smaller than the line width, thereby facilitating the formation of prefabricated circuits with superior electrical properties. Considering Ag ink, with a particle size range of 10–50 nm as an example, it was found that it could effectively get filled into a template with a linewidth of 300 nm, enabling the printing of gate line with a resolution of 300 nm, as shown in Fig. 3a. Furthermore, under the confining effect of the templates, the lateral wetting of the ink was completely eliminated, thus enabling ultra-small-spacing printing with a resolution of 300 nm. The top-view SEM image of the printing results demonstrates the uniform reliability of TCA printing. Moreover, the cross-sectional SEM image shows that the circuit cross-section consists of tightly stacked Ag particles, indicating that the printed linewidth is in proximity to the printing limitation. Figure 3b demonstrates the reliable versatility of the TCA printing technique for high-resolution printing on a wide range of materials. The cross-sectional SEM images of large-area grid lines, printed with a linewidth of 1 μm, showcase the consistent thickness of the printed features, demonstrating the precise control over the linewidth and spacing of the designed lines. Furthermore, well-defined gate lines composed of MWCNTs, titanium dioxide (TiO_2_), and P(VDF-TrFE) materials with a line width of 1 μm were successfully printed, demonstrating the ability to print multiple materials at high resolution.

In addition to the higher resolution, the fabrication process resulted in spatially accurate printing of the circuits due to the fact that the functional patterning remained within the template throughout the transfer printing process and there was no excess pressure. By analyzing a typical grid line circuit and taking the accuracy distortion between the master mold and the printed circuit as a reference, the obtained spatial position accuracy was found to be below 50 nm (Fig. S11), which provides a guarantee for applications in some heat dissipation or optical structures.

Furthermore, the thickness of circuits printed by TCA printing was controlled by adjusting the solid content of the ink material, the number of fills, or the template depth. For instance, by utilizing a template with a line width of 8 μm, a depth of 12 μm, and Ag ink with 30% solid content, circuits with Ag-particle thicknesses of 3, 6, 9, and 12 μm were achieved, as shown in Fig. 3c. The templates utilized in TCA printing offer the distinct advantage of not only precisely controlling line width and cross-sectional morphology, but also facilitating the confinement of multiple layers, thereby enabling the fabrication of self-aligned multilayers. The results of printing multiple materials and multilayers with UV adhesive as the base layer are shown in Fig. 3d. The results demonstrate the successful printing of a single layer of MWCNTs, a bi-layer of AgNPs and MWCNTs, another bi-layer of MWCNTs and PVA/H_3_PO_4_, and a triple-layer of MWCNTs, PVA/H_3_PO_4_, and MWCNTs, respectively. In these prints, the heterogeneous materials were strongly bonded because the interpenetration of the two materials at the bonding interface promoted mechanical interlocking.

TCA printing technology employs prefabricated templates to define patterns, which simplifies the printing of precise and complex morphologies. Figure 3e illustrates different morphologies of printed arrays of dots, lines, spirals, and meshes, characterized by well-defined edges and exceptional precision. Moreover, the TCA printing technique can easily achieve conformal printing of cross-scale linewidth patterns. Considering the MWCNTs material as an example, patterns with feature sizes ranging from 20 μm to 4 mm were successfully printed on the surface of a glass sphere with a diameter of 40 mm (Fig. S12).

### Versatility of printing technology

**Fig. 4:**
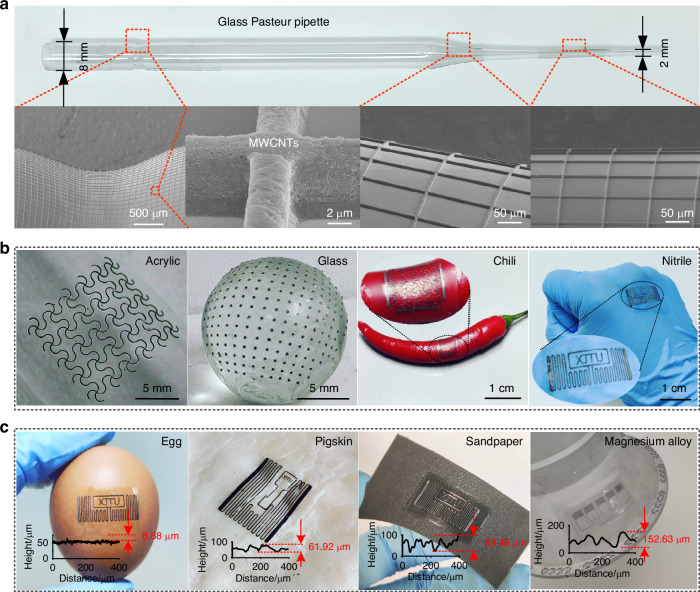
Demonstration of the ability to print circuits on multiple substrates. **a** Optical image of circuits printed on the surface of a glass Pasteur pipette with significant curvature. The partially enlarged SEM image of the printed circuit at three different curvature positions. **b** Demonstration of circuits printed on smooth substrates (acrylic, glass, chili, and nitrile gloves). **c** Demonstration of circuits printed on rough surfaces (egg, pigskin, sandpaper, and magnesium alloy). Insets: Roughness characterization of these substrate surfaces

One of the biggest advantages of TCA printing lies in its remarkable flexibility, enabling the fabrication of high-precision electronics on substrates of virtually any shape or roughness, provided that the soft template should conform to the substrate surface. Figure 4a exhibits that MWCNTs mesh circuits were successfully printed on the surface of a glass Pasteur pipette with end diameter of 8 and 2 mm. Furthermore, the mesh circuits were printed on the saddle, conical, and cylindrical surfaces of the substrate, and the inserted SEM image is a partial magnification of the printed circuits at the three locations. The precision exhibited by the meshes printed at non-regular locations underscores the high fidelity of TCA printing technology and its adaptability toward substrates with arbitrary shapes. Moreover, structures with multiple morphologies can be printed on arbitrary substrates, such as curved meshes, arrays of dots, RFID antennas, and arrays of interdigital electrodes. These substrates include smooth surfaces such as acrylic, glass, chili peppers, and nitrile gloves; and rough surfaces such as egg shells, pigskin, sandpaper, and magnesium alloys. Figure 4b showcases circuits printed on smooth substrate surfaces; that is, a curved mesh of AgNPs with a line width of 200 μm on an acrylic cylindrical surface with a diameter of 2 cm; and arrays of MWCNT dots with a 200 μm diameter on glass spherical surface with a diameter of 1 cm. The results of these two printed structures demonstrate that the TCA printing technique can be adapted to expandable or non-expandable substrates, with designed continuous or discontinuous morphology. Notably, one of the key advantages of TCA printing technology lies in its exceptional flexibility to print graphics on virtually any existing substrate, exemplified by the printing of AgNP meshes on a chili pepper or an RFID antenna made of AgNP material on the back of a hand covered in a nitrile glove.

Unlike traditional transfer printing technologies that rely on surface energy, TCA printing technology does not impose restrictions on the type of receiving substrate. It can be used to print electronic circuits even on rough surfaces and achieve conformal and robust circuits. The circuit printed on a rough substrate is shown in Fig. 4c, where an RFID antenna is printed on the surface of an eggshell with a roughness of 8.68 μm, demonstrating the convenience of TCA printing technology in printing high-precision graphics on the surfaces of non-flat, non-expandable hard substrate. Furthermore, the strong adaptability of TCA printing techniques to flexible and rough substrates is demonstrated by the fabrication of antennas on bio-pigskin surfaces with a roughness of 61.92 μm and sandpaper surfaces with a roughness of 89.48 μm. Moreover, TCA printing technology provides a flexible method for manufacturing surface circuits on components of large-scale equipment, offering significant implications for enhancing equipment intelligence through the integration of surface circuits. For example, the precise printing of arrayed interdigitated electrodes on the surface of magnesium alloy components (with a roughness of 152.63 μm) enables the fabrication of sensing units for pressure, temperature, strain, and other applications, demonstrating practical significance in intelligent device components.

### Conformal temperature/humidity sensor

The integration of conformal thin-film circuits on device surfaces represents a promising approach for developing sensors that are pivotal in advancing the equipment intelligence. Although previous technologies have often involved the attachment of flexible electronics to equipment surfaces^47^ to manufacture high-performance conformal electronic components, the flexible substrate carrying the electronics brings forward new challenging problems such as insufficient adhesion and limitations in adapting to complex environmental conditions (e.g., low or high temperatures, scratches), thus rendering them unsuitable for extreme service environments. Direct printing of circuits on component surfaces emerges as a more viable strategy for integrated electronics manufacturing. In this study, circuits were printed on the surface of a ceramic vase via TCA printing technology, and then well-established electronic components were assembled on its surface to fabricate a conformal temperature and humidity sensing system. Further, relevant functional tests were conducted to provide a reference for fabricating integrated circuits on the surface of the device.

**Fig. 5:**
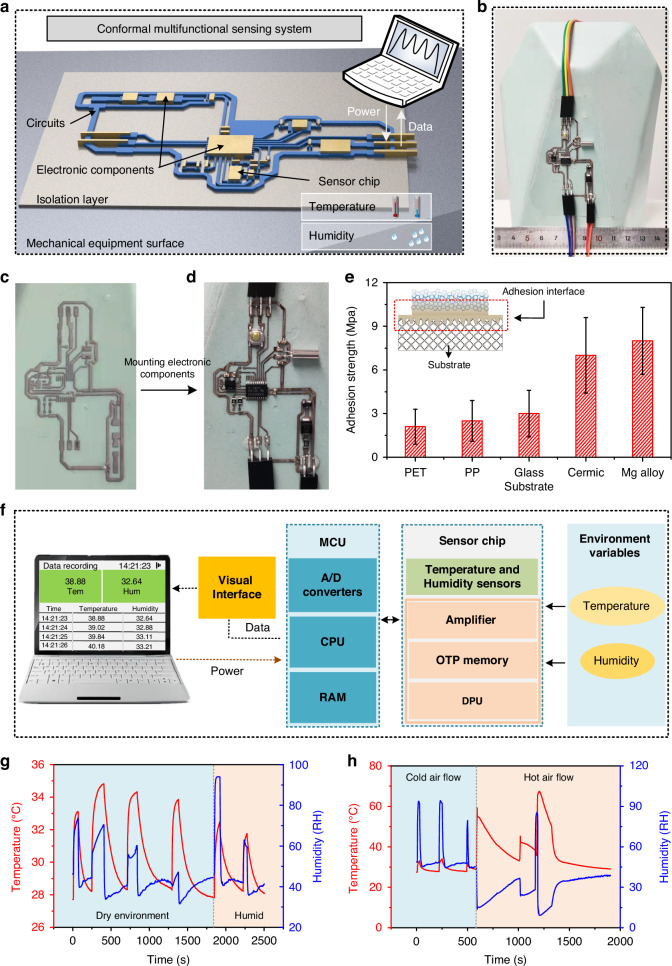
Conformal multifunctional sensing system. **a** Schematic diagram of the integrated temperature and humidity sensing system on the surface of the device. **b** Optical image of the temperature and humidity sensor system fabricated on the surface of a ceramic vase. **c** The printed circuits, and **d** soldered electronic components. **e** Peel strength of TCA printed AgNP circuits on different substrates. **f** Block diagram of the sensing principle of the conformal temperature and humidity sensing system. **g** The test results of the parts in a dry and humid environment. **h** The test results of the parts under cold and warm air currents

The schematic of the designed surface conformal multifunctional sensing system is shown in Fig. 5a, where an isolation layer and a sensing layer are overlaid on the surface of the equipment, with the sensing layer consisting of circuits and electronic components. This system enables efficient data acquisition, signal processing, and information transmission of the temperature and humidity conditions of the environment where the part is situated. A computer system was utilized for powering the system, as well as for receiving, processing, and displaying the sensory outputs. Highly precise circuits boards were seamlessly integrated onto the device surface with ultra-thin profiles and superior adhesion properties, thereby enabling the tight integration of printed sensing electronics with minimal impact on the device forms and weight. The optical images of the conformal temperature and humidity sensing system fabricated on the surface of a ceramic vase are shown in Fig. 5b, the schematic diagram of the electrical temperature/humidity sensing unit is shown in Figure S13, and the component layout is shown in Figure S14.

First, the Ag circuits were printed on the surface of the ceramic vase according to the pre-defined pattern (Fig. 5c), and then the sensor chip, microprogramming control unit (MCU), resistors, capacitors, and other electronic components were assembled onto the circuits by surface mounting technology (Fig. 5d), and finally packaged. The TCA printing technology is not only compatible with ceramic substrates, but also shows versatility across PET, PP, and glass substrates (Figure S15). To confirm the potential of the fabricated conformal circuits in harsh environments, the adhesion of printed circuits on various substrates was tested by the pull-out method, and the results are shown in Fig. 5e. In circuits printed on substrate, the UV adhesive serves as a connecting layer between the functional material of the circuit and the substrate, establishing close contact with the substrate and forming a deep interlock with the functional material. During adhesion testing by the pull-off method, the bonding strength between the circuit and the UV adhesive layer typically surpasses that between the UV adhesive and the substrate, resulting in the separation of interface predominantly at the binding interface between the substrate and the UV adhesive. The adhesion strength is directly influenced by the surface roughness and surface energy of the substrate. Test results indicate that the adhesion strengths of circuits on PET film, PP plastic, glass, ceramic, and magnesium alloy surfaces are 1.8, 2.1, 3.0, 6.8, and 8.1 MPa, respectively. The robust adhesion provides a solid foundation for the circuits to operate effectively in extreme environmental conditions.

The block diagram and working principle of the fabricated conformal temperature and humidity sensing system are shown in Fig. 5f, illustrating the power delivery, data conditioning, and data transmission pathways. The sensor chip contains a bandgap temperature sensor, capacitive relative humidity sensor, amplifier, one-time programmable (OPT) memory, digital processing unit (DPU), etc., which facilitate the conversion of detected temperature and humidity data into a stabilized voltage. The voltage signal converted from the capacitive output of the humidity sensor, and the voltage signal from the temperature sensor, were regulated to a stable voltage signal by signal conditional paths of analog circuits. Subsequently, the MCU integrated with analog to digital (A/D) converters was used to convert all analog signals processed by the sensor chip to digital signals. The central processing unit (CPU) conducted data processing tasks such as filtering, amplifying, and linearizing to enhance data accuracy and reliability. The data were then transferred to random access memory (RAM) and further transmitted to the computer system. Finally, a customized visualization program was developed on the computer system for receiving, processing, displaying, storing, and sharing the digital signals of transmitted temperature and humidity data (Figure S16).

To demonstrate the universality and responsiveness of the fabricated conformal thin film circuits under different conditions, two sets of user-friendly and accessible approaches were presented herein to detect and respond to the circuit’s ability to sense temperature and humidity changes. Noteworthy, in this study, the commercially verified products were used as sensors, and their accuracy was thoroughly verified through extensive testing and validation by the manufacturer (detailed sensor information is presented in Table S3). In the first set of experiments, dry and humid environments were, respectively, established for comparative analysis. The sensor position of the vase was intermittently touched with dry and humid fingers, respectively, and the corresponding results are shown in Fig. 5g. The results show that the system was able to accurately output the values and durations of the temperature and humidity of the environment, which proves that the system has the basic function of sensing temperature and humidity. In the second set of experiments, the comparative analysis of cold airflow and hot airflow environments was conducted, these two environments were emulated using blowers and the test results are shown in Fig. 5h. In the cold airflow environment, the temperature increased slightly while the humidity changed significantly. Conversely, in the hot airflow environment, the humidity decreased while the temperature increased significantly due to the evaporation of water caused by the hot airflow, demonstrating the sensitive temperature and humidity sensing ability. In summary, the fabricated conformal skin temperature and humidity sensing circuit system is capable of sensing and transmitting signals in dry/wet and cold/hot environments, thus reconfirming the robustness of the circuits to maintain stable outputs despite challenging environmental conditions. Moreover, the versatility of robust conformal electronic printing facilitates the development of a variety of applications, such as wearable electronics, conformal antennas, and aircraft skins.

### Conformal ultra-thin energy storage units

**Fig. 6:**
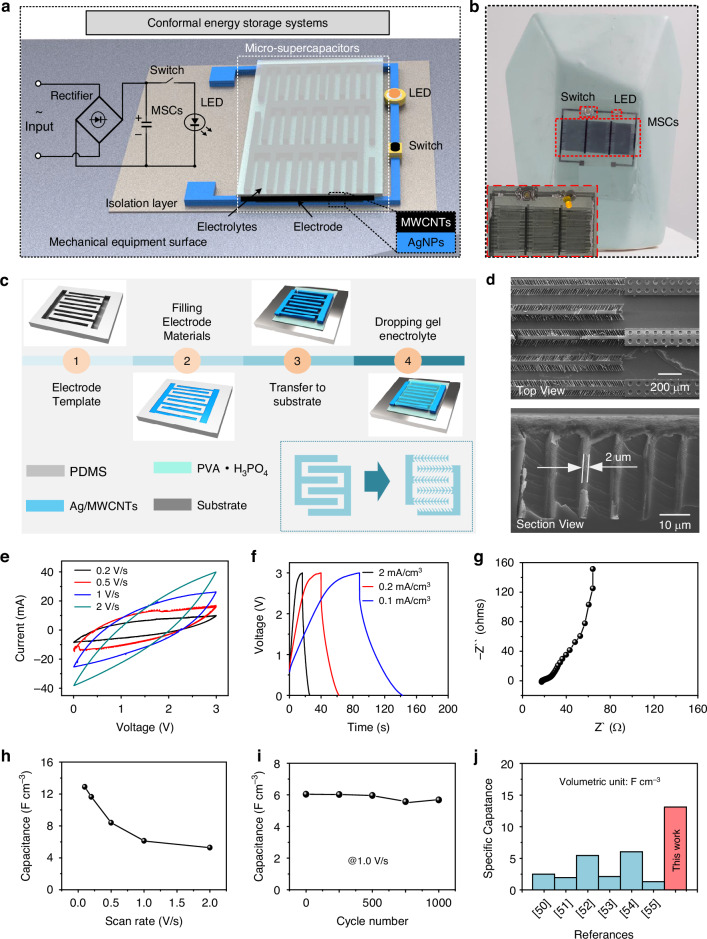
Conformal energy storage system. **a** Schematic and equivalent circuits of the integrated energy storage system on the surface of the device. **b** Optical image of three series-connected interdigitated MSCs systems fabricated on the surface of a ceramic vase. Insert Optical diagram of MSCs lighting up the LED. **c** Fabrication process of the integrated MSCs. Insert: Optimized structure of the interdigital electrode. **d** SEM image of the top view of structured interdigital electrode mentioned above and SEM image showing cross-sectional view below. **e** CV curves of the integrated MSCs at different scan rates from 0.2 to 2 V·s^−1^. **f** GCD curves of the integrated MSCs at different current densities from 0.1 to 2 mA·cm^−3^. **g** Nyquist impedance plots of the integrated MSCs. **h** Capacitance of the integrated MSCs at different scan rates from 0.2 to 2 V·s^−1^. **i** Capacitance over 1,000 charge/discharge cycles. **j** Electrode volume–specific capacitance comparison of different CNT-based MSCs

Micro-supercapacitors have attracted significant research attention because they can be easily integrated with microelectronic devices. They constitute an indispensable part of microelectronic systems by providing a power source for sustainable investigation in areas such as microelectromechanical systems, micro-robotics, and wearable electronics. In recent years, integrated energy storage units have been developed on surfaces with different substrates, such as buildings or scalable surfaces, which has enabled efficient harvesting and utilization of energy^48,49^. However, the fabrication of ultrathin energy storage systems on the surfaces of devices is a challenging task due to the lack of a generalized technology. This study demonstrates the fabrication of a conformal ultrathin skin energy storage system on a device surface, as shown in Fig. 6a. An external charging device converts the incoming AC signal into a DC signal and stores it in the micro-supercapacitors (MSCs), and the stored energy is used to light an LED. The system consists of MSCs and functional cables, where both the electrodes of MSCs and the cable were in-situ fabricated by TCA printing technology; however, their properties are different due to the difference in functions of capacitors and cables. The functional cables exhibit properties such as high-temperature resistance, resistance to mechanical stimulation, etc., as demonstrated in the previous section. The electrodes of the MSCs were optimized to be comb-shaped and were printed by aligning double-layer materials, which led to the improvement in the device performance. The energy storage system fabricated on the surface of a ceramic vase is shown in Fig. 6b, which consists of MSCs formed by three series-connected electrodes, a switch, an LED, and the interconnection cable. The inset shows an optical image of the system lighting up an LED.

The fabrication process of the conformal MSCs is shown in Fig. 6c. The electrode materials were filled in the flexible PDMS templates and printed on the substrate (the double-layer material was printed, in which AgNPs were used as the collector and MWCNTs were used as the electrodes). The thickness of the printed AgNPs collector was about 5 μm, and that of the electrode MWCNTs was about 35 μm. Thus, an ultrathin, conformal interdigital electrode cell with a total thickness of about 40 μm was obtained. The active electrolyte material PVA/H_3_PO_4_ was then poured onto the interdigital electrodes to form an integrated conformal MSC. The preparation process of PVA/H_3_PO_4_ is provided in the Methods Section. To improve the performance of the conformal energy storage system, more active electrolyte material must be immersed in the unit volume of the electrodes. Therefore, herein, each strip electrode of the interdigital electrodes was optimized into comb-shaped electrodes to promote sufficient contact between the electrolyte material and the electrodes. The inset diagram shows a schematic of the optimized electrode structure. The top-view and cross-sectional SEM images of the optimized comb-shaped electrodes are shown in Fig. 6d, revealing that the comb-shaped electrode line width is 2 μm, which ensures sufficient contact between the electrolyte and the electrode.

Figure 6e–j demonstrate excellent electrochemical performance of the integrated MSCs. The measurements and calculations are provided in the Methods Section. Figure 6e shows the cyclic voltammetry (CV) curves at different scan rates from 0.2–2 V·s^−1^, exhibiting typical charge/discharge behavior, and the voltage window is limited to 3 V for stable operation. Figure 6f shows the galvanostatic charge/discharge (GCD) curves at different current densities, and the near-ideal triangular GCD curves confirm the ideal capacitance behavior. The cross-axis intercept of the real-axis intercepts of the Nyquist diagram indicates the ohmic resistance of the electrolyte and electrode materials, which matches with IR drop of the GCD discharge curves. The typical electrochemical impedance spectroscopy (EIS) curve exhibits an apparent linear increase with an equivalent series resistance of 18 Ω at low frequencies (Fig. 6g). The volumetric capacitance of the integrated MSCs was calculated from the CV curves at various scan rates by considering the volume of both electrodes and available space. Figure 6h shows that the volume capacity of MSCs is 13.1 F·cm^−3^ at a scan rate of 0.2 V·s^−1^ and can reach 5.8 F·cm^−3^ even at a significant scan rate of 2 V·s^−1^, showing excellent rate capability of the device, which can be attributed to the high performance of the structured electrodes. Furthermore, the cyclability of the MSCs is another essential feature of the device, as the MSCs have a stable volume capacitance after 1,000 cycles at a rate of 1 V·s^−1^ (Fig. 6i). The MSCs prepared with optimized interdigital electrodes demonstrate excellent performance, which make them competitive in integrated devices with volumetric capacitance exceeding the performance of general typical MSCs (Fig. 6j and Supporting Information Tables S4)^50–55^. Incidentally, in this system, although the printed cables and electrodes can withstand temperature and mechanical stimulation, the capacitors cannot work in an environment temperature of more than 40 °C for a longer duration due to the limitation of its electrolyte temperature resistance^56,57^. Moreover, owing to the highly narrow electrode spacing (100 μm) of the planar interdigital electrodes, electrode deformation may lead to short-circuiting under mechanical stimulation. Therefore, in practice, supercapacitors should be additionally protected to prevent them from being stimulated by mechanical forces.

## Conclusions

In this study, TCA printing technology was proposed to manufacture high-quality and robust conformal circuits on arbitrary surfaces. By penetrating the adhesive into the functional materials, the mechanical robustness of the circuits improved, allowing them to withstand harsh conditions such as high temperatures, bending, scratching, and abrasion damage. Taking advantage of the confinement effect of templates fabricated by the photolithography process, TCA printing achieved precise edge accuracy with resolution of up to 300 nm and self-aligned printing of multilayer materials. The proposed printing technology can print a wide range of functional materials (as long as they can be filled into the template) into any shape (isolated/interconnected structures), and can construct conformal circuits on receiver substrates with any morphology (smooth/rough, expandable/non-expandable). Furthermore, herein, temperature/humidity sensing systems and ultra-thin energy storage systems were successfully manufactured on ceramic vases, demonstrating their excellent performance. This technology can be used to build innovative devices for various applications such as autonomous vehicles, robotics, and aeronautics.

## Materials and Methods

### Materials

The PDMS curing agent and elastomer were purchased from Dow Corning (USA). The Ag ink (BroadCON-INK) was purchased from Beijing Dahua BroadTeko Intelligent Technology Co., Ltd. MWCNTs were purchased from Jiangsu XFNANO Materials Tech Co., Ltd. The Norland Optical Adhesive 71 (NOA 71) and 89 (NOA 89) were purchased from Beijing Lienhe Communication Technology Co., Ltd. (China). The OrmoStamp was procured from micro resist technology (Germany). The UVDE200 was purchased from YI XIN TECHNOLOGY (China). The photoresist (AZ4620) was purchased from Merck (Germany). PVA and H_3_PO_4_ were purchased from Aldrich (USA).

### Bionic elastic stamp preparation

Step 1: Double-sided exposure process to manufacture the mold. A layer of metallic Cr was deposited on the glass, which was then spin-coated with photoresist AZ4620 at low speed of 500 rpm for 9 s and high speed of 1,000 rpm for 50 s. Then, it was pre-baked at 95 °C on a baking table for 10 min, and then exposed to 365-nm UV light for 40 s on the front side and 8 s on the reverse side. Finally, it was placed on NaOH development to form a mold with micro-suction cup structure. Step 2: PDMS 184 prepolymer was poured on the mold, and then the soft stamps prepared in advance were pressed on its top. Maintaining the pressure, it was cured in the oven at 95 °C for 1 h, then the mold was removed, and the micro-suction cups were formed on the soft stamp.

### Detailed process of TCA printing

Figure 1b illustrates a detailed explanation of the TCA printing technology, as follows:

Filling of functional materials: the PDMS flexible template was filled with slurry-based functional materials, then the filled materials were evaporated(some materials need to be sintered after the evaporation process) to form the functionalized circuits. (PDMS flexible patterning template formed by remolding of silicon master molds). ii) Filling adhesive: UV adhesive was filled into the graphical channel template, allowed to penetrate the functional material, and subsequently cured under a UV light source (365 nm, 1,500 Mw, 90 s). iii) Spray adhesive and transfer template: UV adhesive was sprayed on the receiving substrate and an elastic bionic stamp was used to transfer the template with functionalized circuits to the receiving substrate. iv) Conformal contact: the template was pressed onto the receiving substrate using elastic stamps to form a conformal contact with the substrate. v) Release of the template: the elastic bionic stamp released the template (infiltrated alcohol could weaken the adhesion of the elastic stamp and enabled the release of the template, the principle is shown in Figure S4). vi) UV curing: the substrate was placed under a UV light source (365 nm, 1,500 Mw) for 120 s to cure the UV adhesive. vii) Template removal: the template was transferred away using an elastic bionic stamp and the functionalized pattern was transferred to the receiving substrate surface.

### Printing of multilayer materials

Material A was filled into a flexible PDMS template, where the ink uniformity was promoted through standard mechanical operations and strict control of ink volume. Subsequently, the solvent was evaporated, which resulted in the uniform deposition of Material A within the template. Material B was then filled in by the same method, and after evaporation of the solvent, Material B was deposited onto Material A. This process was repeated layer by layer for the deposition of subsequent materials. Next, the UV adhesive was filled into the template and allowed to penetrate the functional materials (or when it was in complete contact with the functional material). Subsequently, the UV adhesive was cured for 5 min under a UV light source (365 nm, 1500 Mw). Finally, UV adhesive was sprayed onto the receiving substrate (which could be ozone-treated to enhance adhesion). The template was then placed over the substrate, and the UV adhesive was cured for the second time under UV light. During this curing process, the adhesive cured in the template for the first time and the adhesive sprayed on the substrate underwent tight bonding (as they were the same material). Next, the PDMS template was removed (the low surface energy of the PDMS template made it easy to separate from the functional material without adhesion to the template) and the functional material inside the template was printed onto the substrate to form a multilayer functional graphic.

### Design of the temperature / humidity sensing circuit board

The electronic components of the sensing board include the temperature/humidity sensor chip (SHT20-TR-1.5KS, Sensirion, Switzerland), microprogrammed control unit (MCU, MSP430G2553IPW20R, Texas Instruments, USA), linear voltage regulator (XC6206P332MR, Youtai Semiconductor, China), and other electronics such as resistors, capacitors, and quartz crystal.

### Preparing electrolyte solutions of PVA/H_3_PO_4_

PVA (1 g, Mw: 95,000 g·mol^−1^) was dissolved in ultrapure water (15 mL) under a water bath at 95 °C and then cooled down to room temperature. H_3_PO_4_ (1.6 g, 85 wt.%) was added to the PVA solution and mixed magnetically at room temperature for 12 h to obtain a homogeneous solution.

### Performance testing of a conformal ultra-thin energy storage system

Measurement: All electrochemical tests, including cyclic voltammetry (CV) and galvanostatic charge/discharge (GCD) studies, were conducted at room temperature on an electrochemical workstation (Versastat 3, Princeton Applied Research, USA). Calculations: The electrochemical performance of the device was evaluated by CV and GCD. The volumetric capacitance (C) was calculated by using the following equations from the CV response curve for a scan rate in the range of 0.2–2 V·s^−1^:

$${C}_{V}=\frac{1}{{\mathcal{V}}\varDelta V}\int I\left.\left(t\right)\right|{dt}$$

where ΔV is the potential range (3 V for three-series connected MSCs), ${\mathcal{V}}$ is the total electrode volume, which includes the volume of both the electrodes and the space between them, I(t) denotes the current measured during CV testing, and t is the time.

## Supplementary information

Supporting Information

Robust characterization of circuitsfabricated by TCA printing technology
