# Supplementary material for: Root-inspired, template-confined additive printing for fabricating high-robust conformal electronics
Source: Microsyst Nanoeng. 2024 Dec 14;10:191. doi: 10.1038/s41378-024-00840-z (PMC11646281; doi:10.1038/s41378-024-00840-z)
Supplement: Supplementary file 1 — Supporting Information [file 41378_2024_840_MOESM1_ESM.docx]

Supplementary information

**Root-inspired, template-confined additive printing for fabricating high-robust conformal electronics**

Guifang Liu^1^, Xiangming Li^1,2*^, Yangfan Qiu^1^, Chuanhang Zeng^1^, Xinkai Zhu^1^, Chao Wang^1^, Xiaoliang Chen^1,2^, Chunhui Wang^1^, Hongmiao Tian^1^, Jinyou Shao^1,2*^

^1^ State Key Laboratory for Manufacturing Systems Engineering, Xi’an Jiaotong University, Xi’an, Shaanxi 710049, China.

^2^ Frontier Institute of Science and Technology (FIST), Xi’an Jiaotong University, Xi’an, Shaanxi 710049, China.

* Corresponding authors at: State Key Laboratory for Manufacturing Systems Engineering, Xi’an Jiaotong University, Xi’an, Shaanxi 710049, China.

E-mail addresses: [xiangmingli@xjtu.edu.cn](mailto:xiangmingli@xjtu.edu.cn); [jyshao@xjtu.edu.cn](mailto:jyshao@xjtu.edu.cn)

The file includes:

Tables S1 to S4

Figures.S1 to S16

Movie S1

**Table S1. Detailed comparison between the TCA printing and others typical transfer printing.**

|  |  | **Ref.1** | **Ref.2** | **Ref.3** | **Ref.4** | **Ref.5** | **Ref.6** | **Ref.7** | **Ref.8** | **TCA Printing** |
| --- | --- | --- | --- | --- | --- | --- | --- | --- | --- | --- |
| **1** | **Stamp type** | Elastomeri  stamp | Nanoporous stamp | Inflated elastomeric balloon | Rubber stamp | Dual-functional bilayer polymer thin film | Thermally reflowable carriers, e.g.sucrose | PDMS template | Laser-driven hierarchical “gas-needles” | Bionic elastic stamp |
| **2** | **Resolution** | 0.3 μm | 3 μm | 1 μm or sub μm | Tens of micrometers | 8 nm | 1 μm | 50 nm | Tens of micrometers | 300 nm |
| **3** | **Edge accuracy** | Nano-scale | Depends on applied pressure | Nano-scale | Micron-scale | Nano-scale | Nano-scale | Nano-scale | Nano-scale | Nano-scale |
| **4** | **Thickness** | From nm to μm | Nano-scale | From nm to mm | Tens of micrometers | From nm to μm | From nm to μm | nm | From μm to mm | From nm to mm |
| **5** | **Materials** | Dry, solid ink, or membranes | Wet, slurry ink | Dry, solid ink, or membranes | Wet, slurry ink | Dry, membranes | Dry, membranes | Dry, solid ink | Dry, chips | Dry, Cured solution-processible materials |
| **6** | **Multiple materials printing** | Multilayer non-aligned assemblies | Multi stamp is required, and the materials is non-aligned | Multilayer non-aligned assemblies | Multi stamp is required, and the materials is non-aligned | Multilayer non-aligned assemblies | Multilayer non-aligned assemblies | Multi-layer self-alignment is theoretically possible | Multilayer non-aligned assemblies | Multilayer self-aligned assemblies |
| **7** | **Bonding mode to the substrate** | Surface energy | Surface energy | Surface energy | Surface energy | Surface energy | Surface energy | Surface energy | Surface energy | Penetrating adhesive bonding |
| **8** | **Mechanical**  **robustness** | Weak | Weak | Weak | Weak | Weak | Weak | Weak | Weak | Strong |
| **9** | **Receiving**  **substrate** | Smooth substrate | Smooth substrate | Smooth substrate | Smooth substrate | Smooth substrate | Smooth or rough substrates | Smooth substrate | Smooth substrate | Smooth and rough substrates |

[1] M. A. Meitl, Z. T. Zhu, V. Kumar, K. J. Lee, X. Feng, Y. Y. Huang, I. Adesida, R. G. Nuzzo, J. A. Rogers. Transfer printing by kinetic control of adhesion to an elastomeric stamp. **Nature Mater** 2006, 5, 33.

[2] S. Kim, H. Sojoudi, H.Zhao, D. Mariappan, G. H. McKinley, K. K.. Gleason, A. J. Hart, Ultrathin high-resolution flexographic printing using nanoporous stamps. **Science advances**, 2016, 2,12.

[3] K. Sim, S. Chen, Z. Li, Z. Rao, J. Liu, Y. Lu, S. Jang, F. Ershad, J. Chen, J. Xiao, C. Yu. Three-dimensional curvy electronics created using conformal additive stamp printing. **Nat Electron**, 2019, 2, 471.

[4] H. Wu, S. W. Chiang, C. Yang, Z. Lin, J. Liu, K.S. Moon, F. Kang, B. Li, C. P. Wong. Conformal Pad-Printing Electrically Conductive Composites onto Thermoplastic Hemispheres: Toward Sustainable Fabrication of 3-Cents Volumetric Electrically Small Antennas. **PLoS ONE**, 2015, 10, 8.

[5] Jeong, J et al. High-resolution nanotransfer printing applicable to diverse surfaces via interface-targeted adhesion switching. **Nat Commun**, 5, 5387 (2014).

[6] G. Zabow, Reflow transfer for conformal three-dimensional microprinting, **Science**, 2022, 378, 894.

[7] We. Zhu, P. F. Satterthwaite, P. Jastrzebska-Perfect, R. Brenes, F. Niroui, Nanoparticle contact printing with interfacial engineering for deterministic integration into functional structures, , **Sci. Adv.** 2022, 8, eabq4869.

[8] F. Chen, M. Gai, N. Sun Z. Xu, L. Liu, H. Yu, J. Bian, Y. Huang, Laser-driven hierarchical “gas-needles” for programmable and high-precision proximity transfer printing of microchips, **Sci. Adv**. 2023, 9, eadk0244.

**Table S2. Physical properties of four typical UV adhesives**

| UV adhesive | OrmoStamp | NOA 89 | UVDE200 | NOA 71 |
| --- | --- | --- | --- | --- |
| Viscosity | 400 cp | 15-20 cp s | 14.5 cp s | 200 cp s |
| Hardness-Shore D | 80 | 40 | 45 | 86 |
| Elastic Modulus | 87000 PSI | 4300 PSI | 14500 PSI | 55000 PSI |

**Table S3. Basic information about the temperature/humidity sensor used in the experiment.**

| Type | SHT20 |
| --- | --- |
| Manufacturers | SENSIRION |
| Typical accuracy of humidity | 3% RH(typical) |
| Typical accuracy of temperature | 0.3 °C(typical) |
| Operating Range of humidity | 0 to 100 RH |
| Operating Range of temperature | -40 to 125 °C |

**Table S4. Volume-specific capacitance comparison of electrodes in different CNT-based MSCs.**

| **Electrodes** | **Electrolytes** | **Scan rates** | **Specific capacitances** | **Ref.** |
| --- | --- | --- | --- | --- |
| CNT | KCl | 1V s^-1^ | 2.3 F cm^-3^ | 50 |
| CNT | PVA/H3PO4 | 1 Vs^-1^ | 1.6 F cm^-3^ | 51 |
| CNT | PVA/H3PO4 | 0.009 A cm^-3^ | 5 F cm^-3^ | 52 |
| CNT | PVA/H3PO4 | 10 mV s^-1^ | 2.02 F cm^-3^ | 53 |
| CNT | PVA/H3PO4 | 20 mA cm^-3^ | 6 F cm^-3^ | 54 |
| CNT | PVA/H3PO4 | 5.1 mA cm^-3^ | 1.2 F cm^-3^ | 55 |
| CNT | PVA/H3PO4 | 1 V s^-1^ | 13.1 F cm^-3^ | This paper |

[50] M. Beidaghi, C. Wang, Micro‐Supercapacitors Based on Interdigital Electrodes of Reduced Graphene Oxide and Carbon Nanotube Composites with Ultrahigh Power Handling Performance, Advanced Functional Materials, 22(2012), 4500.

[51] J. Pu, X. Wang, R. Xu, K. Komvopoulos, Highly Stretchable Microsupercapacitor Arrays with Honeycomb Structures for Integrated Wearable Electronic Systems, ACS nano, 10(2016), 9306.

[52] S. Kim, H. Koo, A. Lee, P. V. Braun, Selective Wetting‐Induced Micro‐Electrode Patterning for Flexible Micro‐Supercapacitors, Adv.Mater. 26(2014), 5108-5112.

[53] L. Liu, D. Ye, Y. Yu, L. Liu, Y. Wu, Carbon-based flexible micro-supercapacitor fabrication via mask-free ambient micro-plasma-jet etching, Carbon, 111(2017), 121.

[54] F. Wen, C. Hao, J. Xiang, L. Wang, H. Hou, Z. Su, W. Hu, Z. Liu, Enhanced laser scribed flexible graphene-based micro-supercapacitor performance with reduction of carbon nanotubes diameter, Carbon, 75(2014), 236.

[55] S. K. Kim, H. Koo, J. Liu, P. V. Braun, Flexible and Wearable Fiber Microsupercapacitors Based on Carbon Nanotube-Agarose Gel Composite Electrodes, ACS Appl. Mater. Interfaces, 9(2017), 19925.


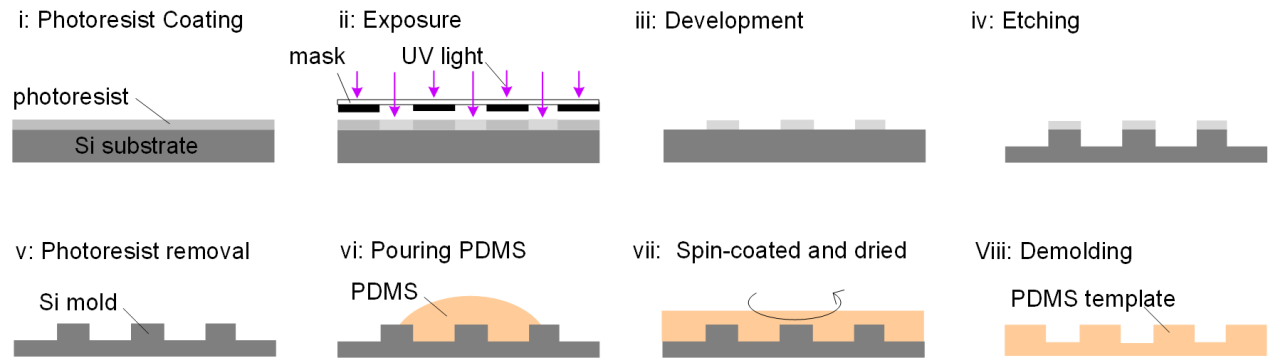


**Figure S1. Preparation of flexible template.** The fabrication of silicon templates, typically employed in standard etching processes, involves a meticulous sequence of operations: Upon a silicon wafer that has been meticulously cleaned and dried, a layer of photoresist is spin-coated and subsequently baked (i). This coated substrate is then subjected to photolithography using a mask imprinted with the intended design (ii). Following exposure, development occurs in a 0.5% NaOH solution, revealing the pattern (iii). A post-exposure bake at 130°C for 30 minutes serves to reinforce the photoresist structure. Etching follows to transfer the pattern onto the silicon surface (iv), after which any residual photoresist is eliminated through washing with an alcohol solution, leaving behind a silicon mold of high precision and defined pattern (v). To produce a soft mold from the silicon template, PDMS (Polydimethylsiloxane), a versatile elastomer, is thoroughly blended with a curing agent. The mixture, after being degassed under vacuum to eliminate air bubbles, is poured onto the silicon mold and subjected to a second degassing cycle (vi). The thickness of the flexible substrate is controlled by adjusting the spin coater speed, which initially operates at 500 rpm for 9 seconds, and subsequently at 1500 rpm for 50 seconds (vii). The final step involves curing the mold at 90°C for an hour on a hot plate, then the PDMS template is uncovered from the silicon mold, culminating in a flexible template with a uniform thickness of approximately 200 μm (viii).


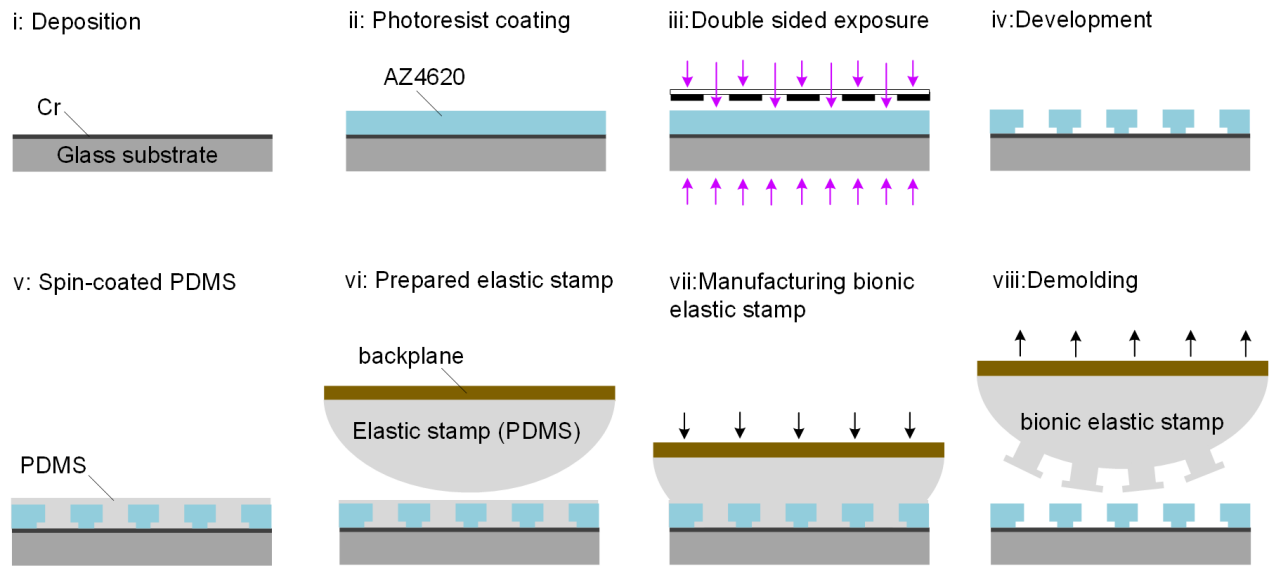


**Figure S2. Preparation of Bionic Elastic Stamps.** i: Deposition of Cr onto the glass substrate to enhance substrate adhesion. ii: Coating with adhesive (AZ4620) and drying. iii: Exposure under UV light source, note that this refers to double sided exposure, front side exposure is for the formation of the through holes, and the back side exposure is for the formation of the end of the bionic adhesive suction cups. iv: Master mold of bionic sucker is formed after developing. v: Pouring PDMS on the master mold and removing air bubbles by vacuum. vi:Preparation of the elastic stamp ( molding by pouring). vii: Pressing of the elastic stamp onto the mold poured with PDMS and curing together. viii: Elastic bionic stamps are formed by demolding after curing.


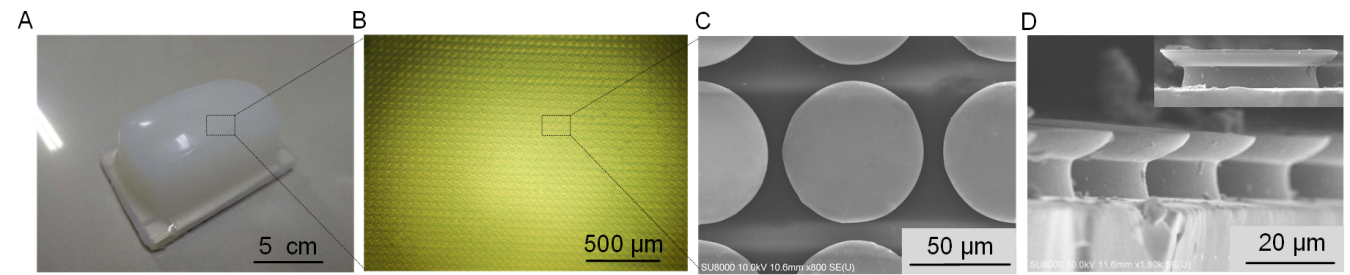


**Figure S3. The manufactured bionic elastic stamp.** (A) Optical image of a bionic elastic stamp.（B）Photoscope image of a large area of microsuckers on the surface of the stamp. （C）Top-view SEM image of microsuckers, and（D）side-view SEM image of microsuckers.


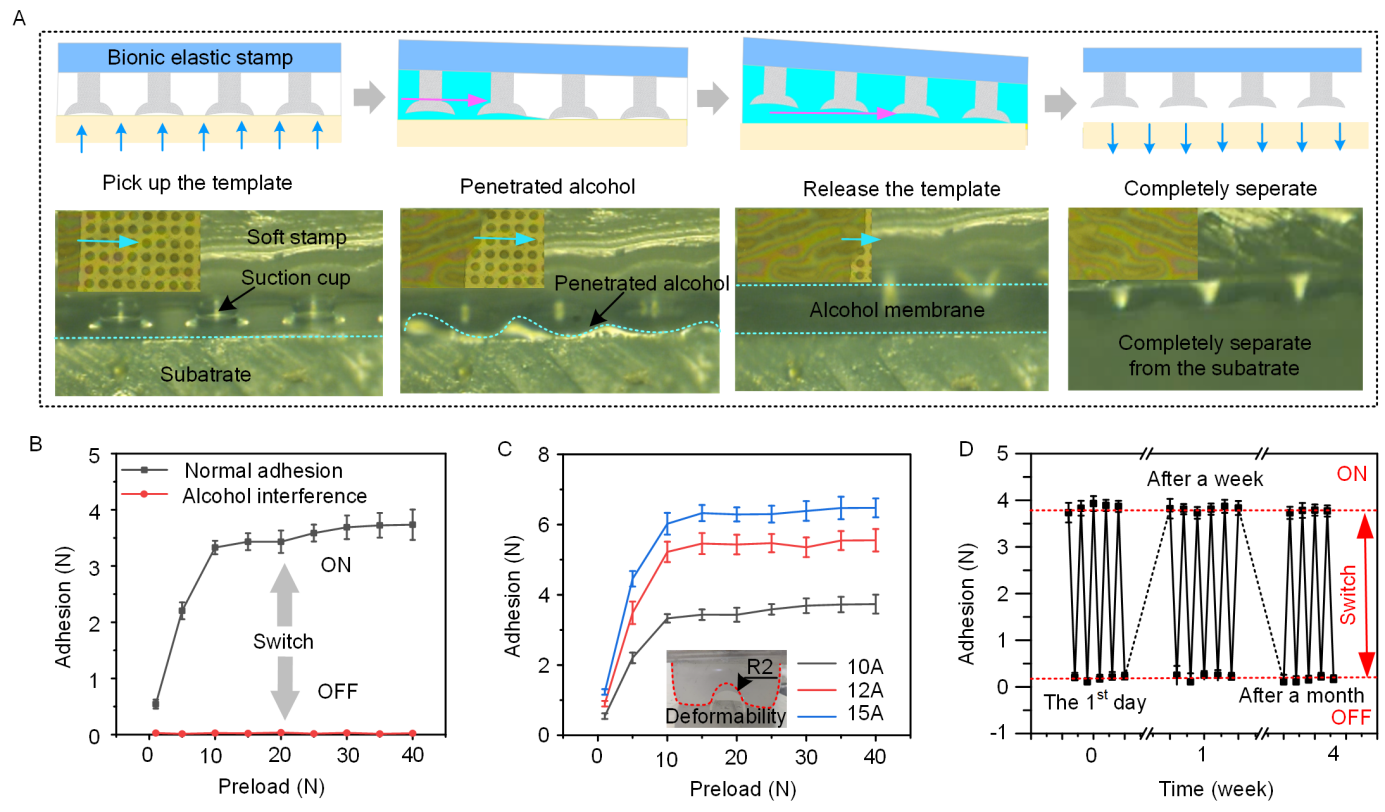


**Figure S4. Characterization of the capabilities of bionic elastic stamps.** (A) Optical images of the detachment process when alcohol penetrates the adhesion interface. (B) Adhesion switching ability of bionic elastic stamps under normal adhesion and alcohol interference. (C) The influence of preloading force on adhesion in stamps with different hardnesses. The inserted image demonstrates the conformability and deformation capability of the bionic elastic stamp on a curved surface with a radius of 2 cm. (D) Adhesion stability of bionic elastic stamps.


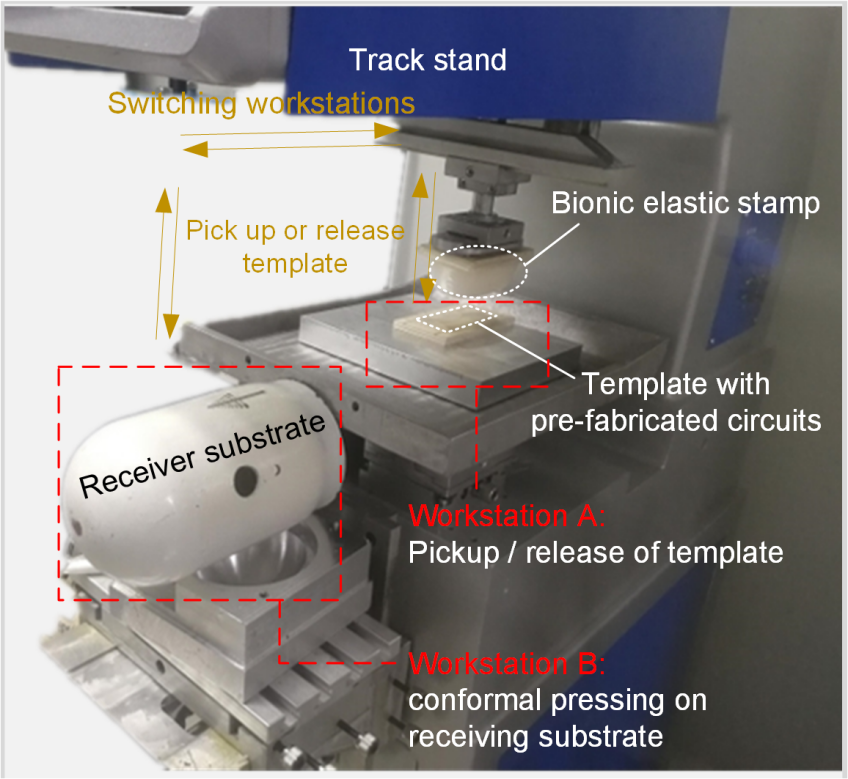


**Figure S5. An optical image of customized TCA printing equipment.**


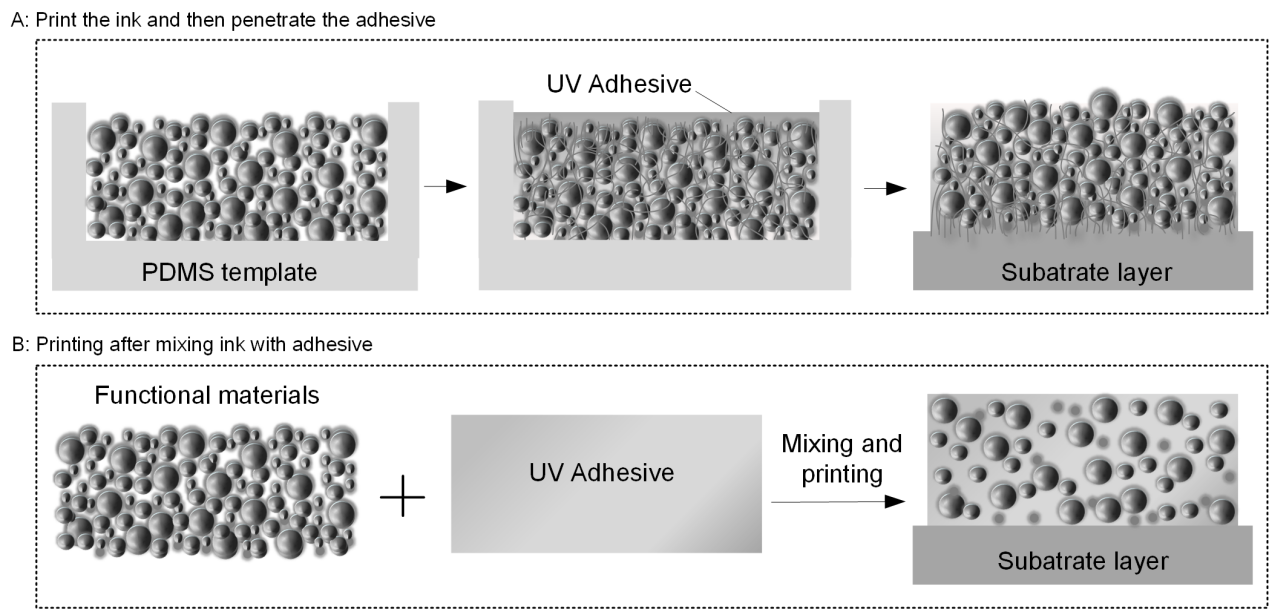


**Figure S6. Comparison of two methods for printing circuits with strong robustness.** (A) TCA printing technique, (B) Simply mixed printing technique.


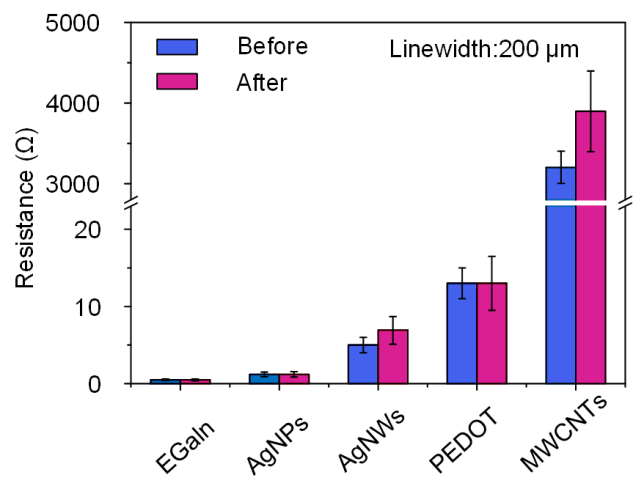


**Figure S7. Comparisons of resistance before and after printing.**

**
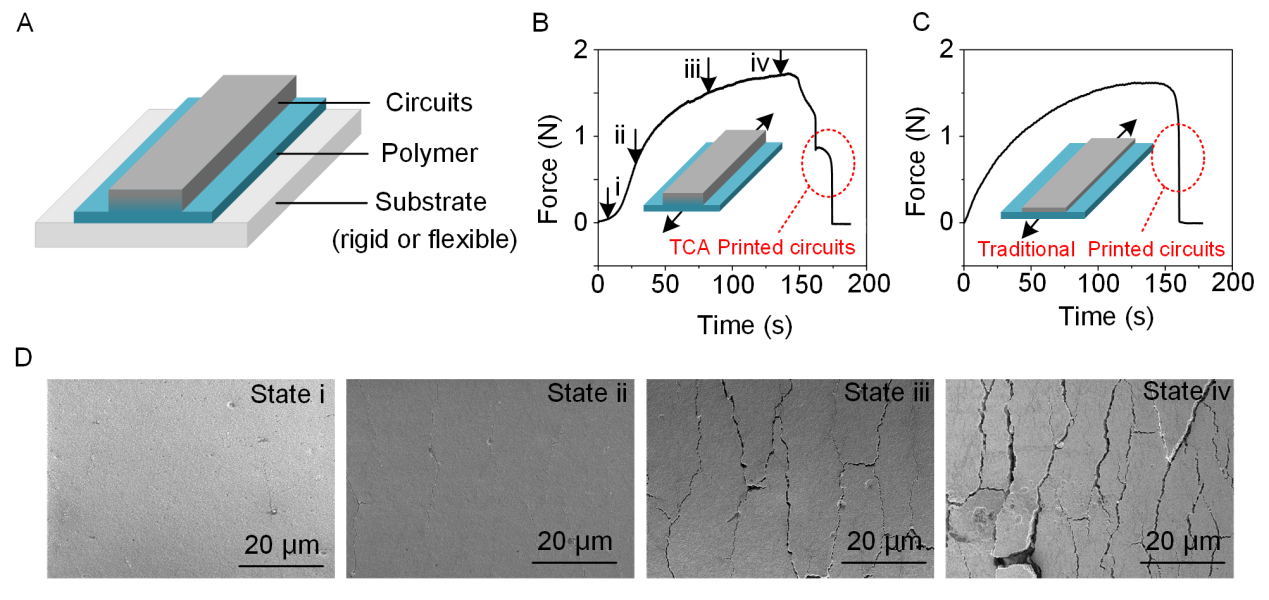
**

**Figure S8. Tensile properties of TCA printed circuits. (A) Schematic diagram of circuit structure. (B) Stretching results of circuits and UV layers fabricated by the TCA printing method (UV adhesive penetrates into the circuit particles). (C) Stretching results of circuits printed on the surface of the UV adhesive layer by the traditional printing method. (D) Cracks in the TCA-printed circuit at different stages of stretching.** Due to the limited tensile properties of the UV adhesive used, the circuit does not have an excellent stretch-rebound ability and can easily be pulled off. As can be seen from the schematic structure of the circuit, the tensile strength of the circuit printed on the substrate is related to both the substrate and the UV adhesive. For non-stretchable substrates, the tensile strength of the circuit depends on the substrate. For stretchable substrates, the tensile strength of the circuit may be determined by the UV adhesive used. When the substrate factor is ignored, the tensile strength of the circuit printed by TCA is better than that of the circuit printed by traditional methods on the UV adhesive layer. The width of the circuit is 0.5 mm, the thickness of the UV adhesive layer is 50 μm, and the speed of the tensile machine is 1 mm/min.


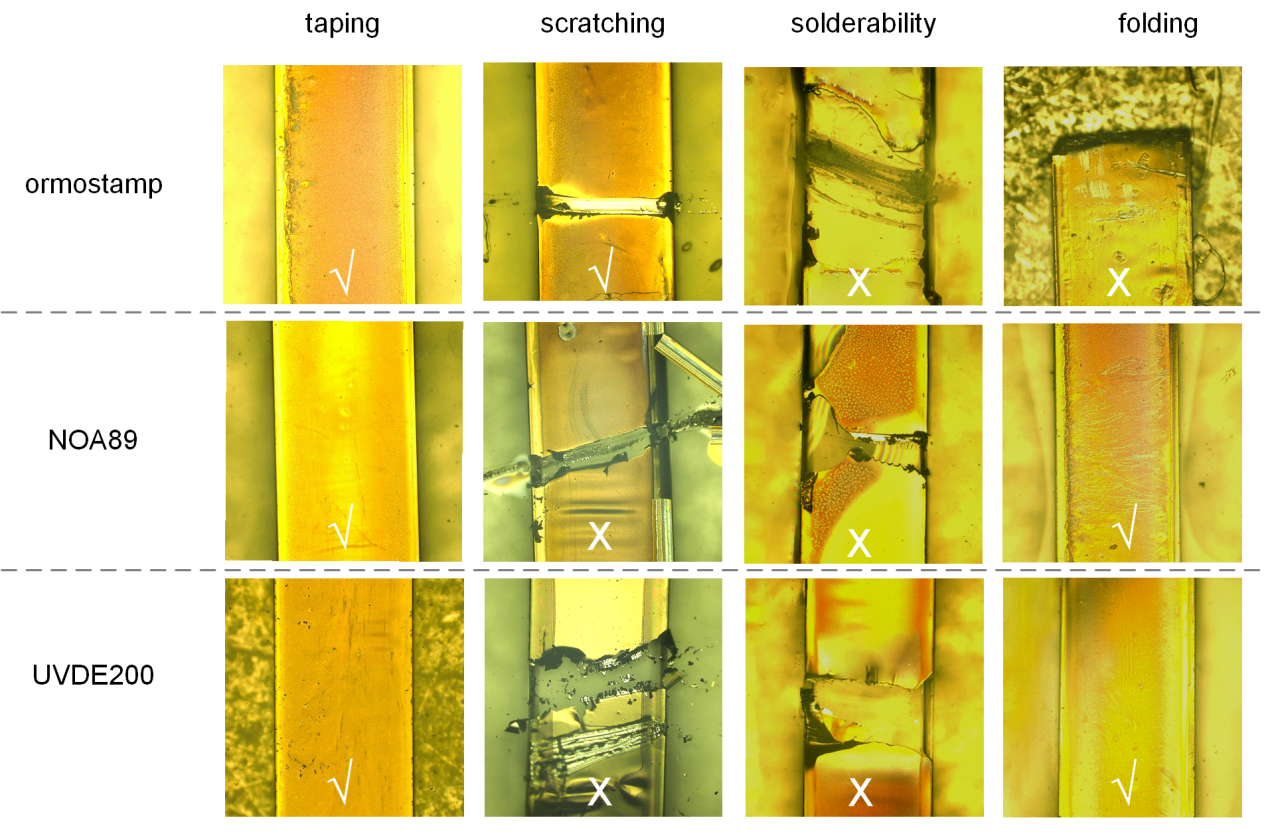


**Figure S9. Results after mechanical loading of circuits printed with OrmoStamp, NOA 89, and UVDE200 UV adhesives, respectively.** **×** means circuits are disconnected, **√** means circuits are connected.


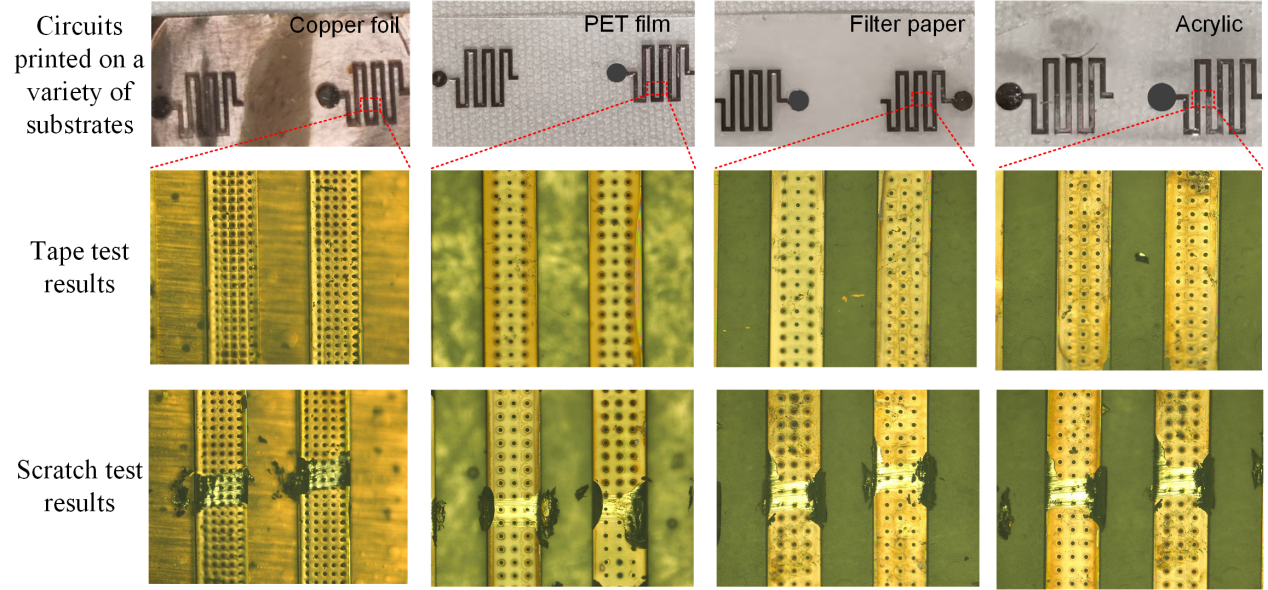


**Figure S10. Tape and scratch test results of circuits printed on different substrates.** NOA71 exhibits excellent adhesion on a variety of substrate surfaces (copper foil, PET film, filter paper, and acrylic plate), and the circuits exhibit excellent electrical connectivity after tape and scratch tests.


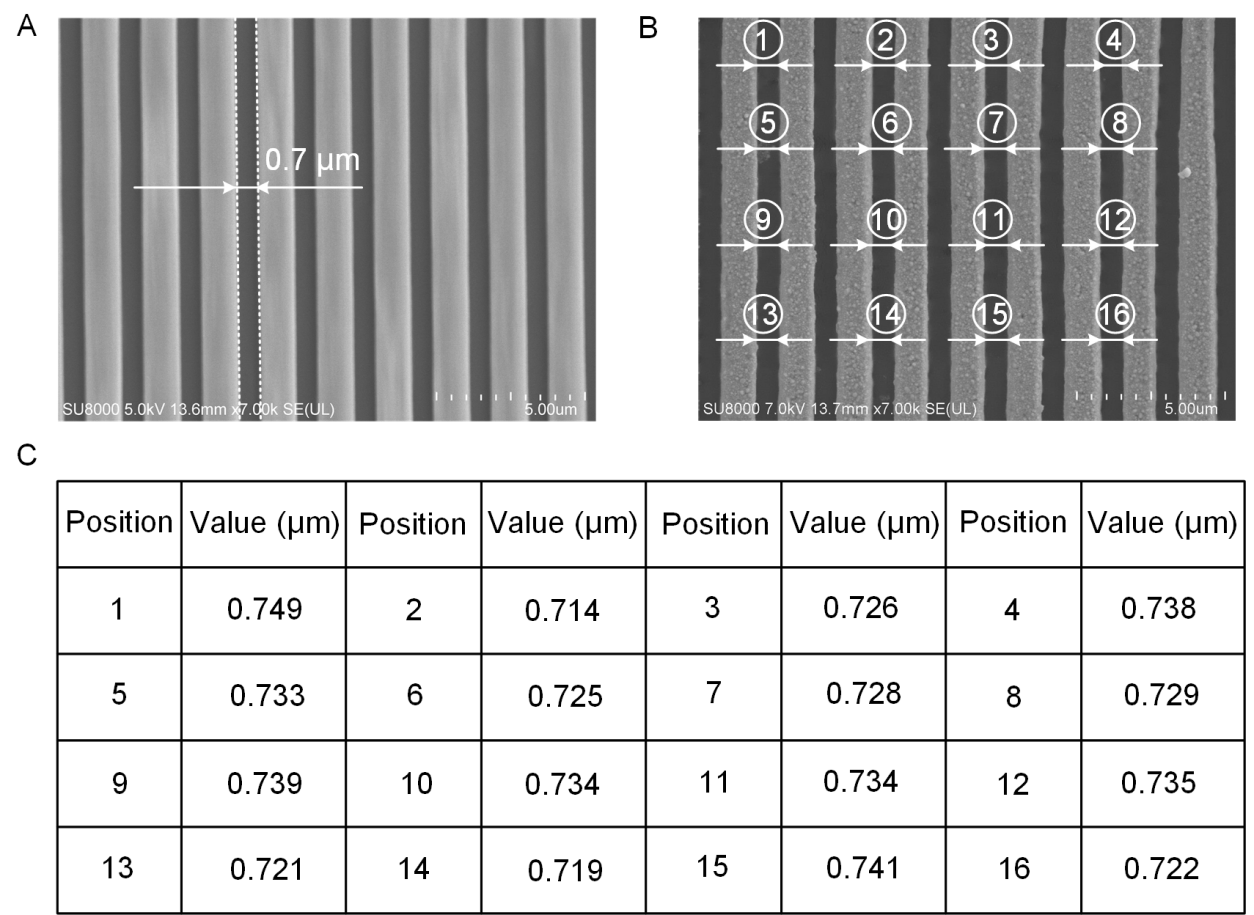


**Figure S11. Quantitative characterisation of spatial printing accuracy of TCA printed circuits.** (A) SEM of the master mould. (B) SEM of circuits printed by TCA technology. (C)The measured spacing values of the circuits at different locations printed by TCA, the spatial distance distortion between the master mould and the printed circuits is less than 50nm.


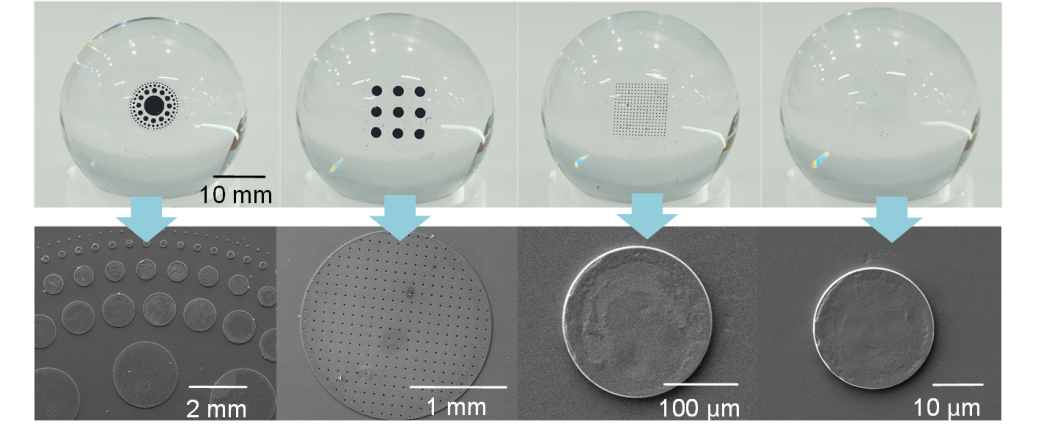


**Figure S12. Images and SEMs of cross-scale graphs printed on glass spheres.**


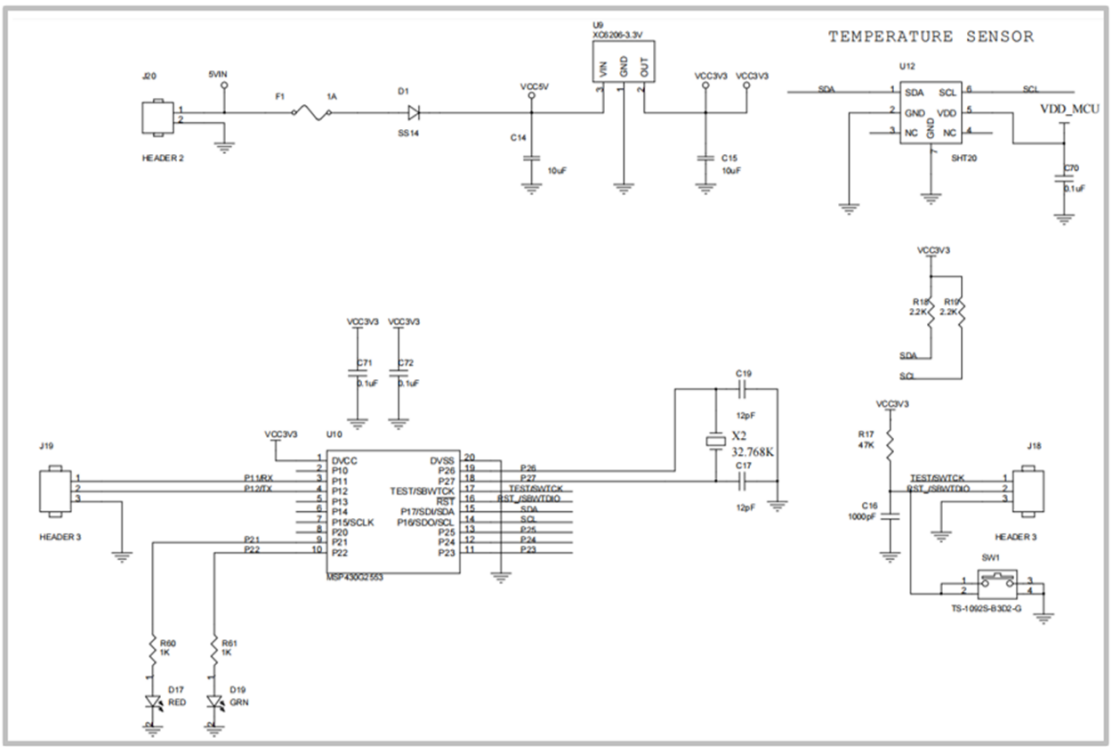


**Figure S13. Electrical schematic diagram of temperature/humidity sensing unit.**


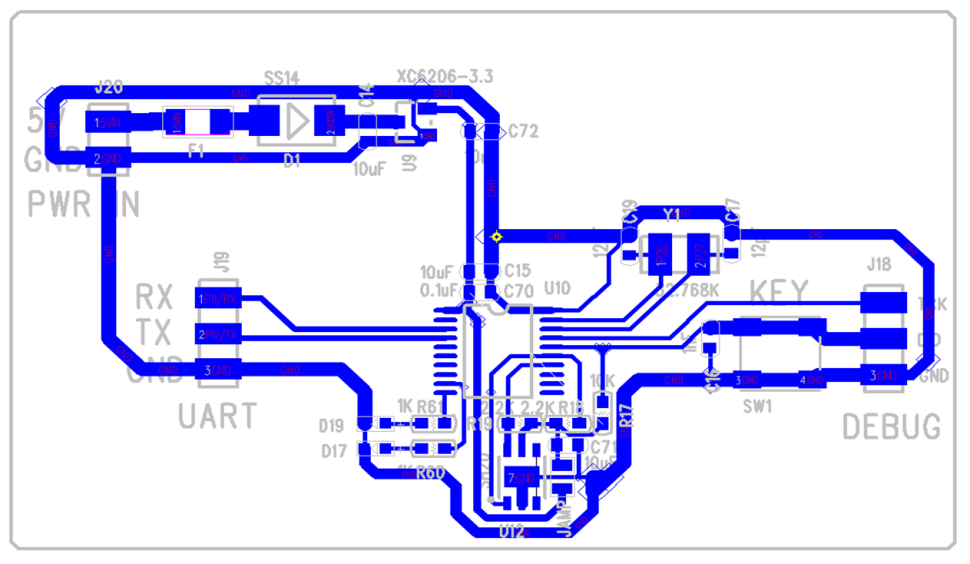


**Figure S14 Component layout of temperature/humidity sensing unit.**


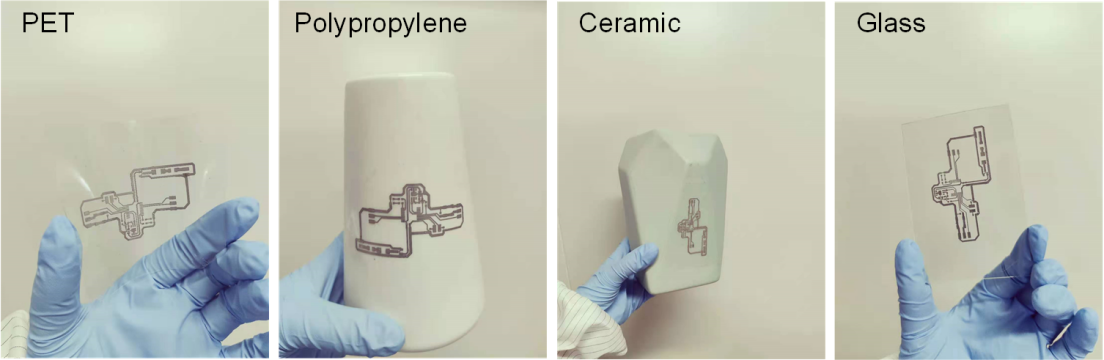


**Figure S15. Images of circuits printed on different substrates.**


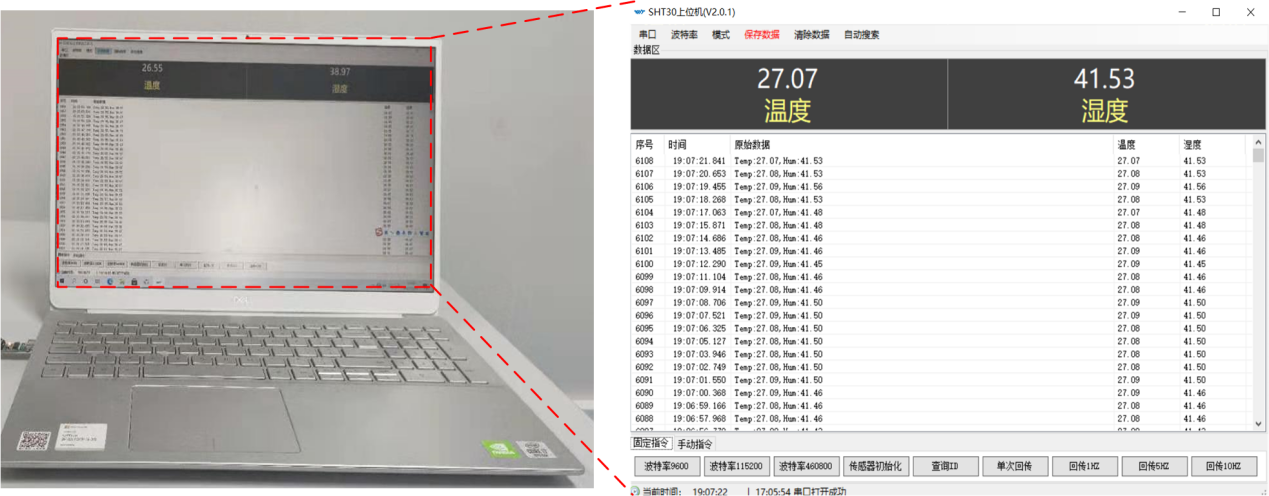


**Figure S16. Customized visualization of the main interface displayed on the computer.**
